# Superinfection of hepatitis A virus in hepatocytes infected with hepatitis B virus

**DOI:** 10.7150/ijms.32795

**Published:** 2019-09-20

**Authors:** Nan Nwe Win, Tatsuo Kanda, Masahiro Ogawa, Shingo Nakamoto, Yuki Haga, Reina Sasaki, Masato Nakamura, Shuang Wu, Naoki Matsumoto, Shunichi Matsuoka, Naoya Kato, Hiroshi Shirasawa, Osamu Yokosuka, Hiroaki Okamoto, Mitsuhiko Moriyama

**Affiliations:** 1 Department of Molecular Virology, Graduate School of Medicine, Chiba University, Inohana 1-8-1, Chuo-ku, Chiba 260-8677, Japan;; 2 Division of Gastroenterology and Hepatology, Department of Medicine, Nihon University School of Medicine, 30-1 Oyaguchi-Kamicho, Itabashi-ku, Tokyo 173-8610, Japan; kanda.tatsuo@nihon-u.ac.jp (T.K.);; 3 Department of Gastroenterology, Chiba University, Inohana 1-8-1, Chuo-ku, Chiba 260-8677, Japan;; 4 Division of Virology, Department of Infection and Immunity, Jichi Medical University School of Medicine, 3311-1 Yakushiji, Shimotsuke, Tochigi 329-0498, Japan

**Keywords:** HAV, HBV, dual infection, miso extracts, rice koji

## Abstract

Hepatitis A virus (HAV) infection is a major cause of acute hepatitis including acute liver failure. Hepatitis B infection (HBV) occurs worldwide, with the highest rates in Asian and African countries, and there are several reports that HAV infection may have a more severe clinical course in patients with chronic HBV infection. We previously demonstrated that Japanese miso extracts have inhibitory effects on HAV replication. In the present study, we examined the replication of HAV and HBV in a hepatocyte superinfection model and the inhibitory effects of Japanese miso extracts on both viruses. According to the results, HAV infection inhibited HBV replication in superinfected hepatocytes, and Japanese rice-koji miso extracts had inhibitory effects on HAV replication. Our findings provide useful information for clinicians in managing HAV infection in patients with chronic HBV infection.

## Introduction

Acute hepatitis A virus (HAV) infection occasionally causes liver failure in patients who have chronic liver diseases 1-3. Although HAV infection often occurs in childhood, resulting in higher anti-HAV rates in developing countries, HAV also infects middle-aged men and women in developed countries and may lead to acute liver failure 4.

Hepatitis B infection (HBV) occurs worldwide, with the highest rates in Asia and Africa 5; however, superinfection of HAV with HBV may be a remaining health problem even with improvement in these regions. Previous research has shown enhanced propagation of HAV in the human hepatoma cell line PLC/PRF5, which has HBV genomes 6, and it has been reported that interferon treatment inhibits expression of HBV envelope proteins in PLC/PRF5 cells 7. Thus, HAV may interfere with HBV replication in chronic HBV-infected patients.

We recently reported that PXB cells, which are human hepatocytes from a severe, humanized, combined immunodeficiency albumin promoter/enhancer-driven-urokinase-type plasminogen activator mouse model, are permissive to both HAV and HBV 8,9. In the present study, we examined superinfection of HAV in PXB cells infected with HBV. We also infected HepG2.2.15 cells with HAV to monitor both viruses and examined the inhibitory effects of Japanese rice-koji miso on the viruses.

## Materials and methods

### Cell cultures and miso extracts

Human hepatoma HepG2 and HepG2.2.15 cell lines 10 were grown in Roswell Park Memorial Institute (RPMI) 1640 medium (Sigma-Aldrich, St. Louis, MO, USA) supplemented with 1%-10% fetal bovine serum (FBS, Thermo Fisher Scientific, Yokohama, Japan) and 1% penicillin/streptomycin (Thermo Fisher Scientific) in a 5% CO_2_ atmosphere at 37°C. HepG2.2.15 cells are derived from HepG2 cells and produce infectious HBV genotype D 10.

Human hepatocyte PXB cells were purchased from Phenix Bio, Higashi-Hiroshima, Japan, and grown in Dulbecco's modified Eagle's medium (DMEM) (Sigma) supplemented with 2% FBS, 20 mM of 4-(2-hydroxyethyl)-1-piperazineethanesulfonic acid (HEPES), 44 mM sodium bicarbonate (NaHCO_3_), 15 μg/mL L-proline, 0.25 μg/mL insulin, 50 nM dexamethasone, 5 ng/mL epidermal growth factor (EGF), 0.1 mM ascorbic acid 2-phosphate (Asc-2P) and 2% dimethyl sulfoxide (DMSO) [2% DMSO-supplemented hepatocyte clonal growth medium (dHCGM)] at 37 °C and 5% CO_2_, as previously described 8.

Japanese rice-koji miso, Kurasaigetsuusujiomiso (KU) (Ando Brewery, Kakunodate, Japan) is prepared from rice (Kitauramura, Akita, Japan), soy (Akita, Japan), and salt with special Yurara yeast (Akita, Japan) 9. Miso extracts were prepared as previously described 9, and the supernatant was then filtered through a 0.45 µm membrane (IWAKI Glass, Shizuoka, Japan).

### Infection of HepG2 and HepG2.2.15 with HAV

HepG2 and HepG2.2.15 cells were seeded in 6-well plates at a density of 1.0 x 10^5^ cells/well. After 24 hours, the cells were washed twice with phosphate-buffered saline (PBS) and infected with the HAV HA11-1299 genotype IIIA strain 9 at a multiplicity of infection (MOI) of 0.01 in RPMI supplemented with 2% FBS. After 48 hours of infection, the cells were washed twice with PBS, followed by exchange of the same medium. After 96 hours of infection, total cellular RNA was extracted for further analysis. Virus growth was measured using a fluorescent-focus-forming assay of serial dilutions of the HAV stock, and the MOI was calculated 11.

### HAV infection of PXB cells with or without HBV infection

Approximately 4.0 x 10^5^ PXB cells/well were inoculated with HBV genotype C at 5 genome equivalents (GEq) per cell in dHCGM medium 8. After 5 days, the cells were infected with the HAV HA11-1299 genotype IIIA strain at an MOI of 0.01 9. On days 1 and 6 post-HBV infection, the media were exchanged with 500 µL of fresh dHCGM. Eight days after starting incubation, the levels of HAV and HBV DNA in the inoculated cells were determined using real-time PCR.

### RNA extraction and quantitation of HAV RNA and HBV DNA

Total cellular RNA and DNA were extracted using an RNeasy Mini kit (Qiagen, Tokyo, Japan) according to the manufacturer's instructions. PCR amplification of the polymerase/envelope protein-coding region of HBV DNA was performed using the sense primer 5'-GTGTTACAGGCGGGGTTTTT-3' and antisense primer 5'-ATAAAACGCCGCAGACACAT-3'. cDNA was synthesized using the Prime Script RT reagent (Perfect Real Time; Takara, Otsu, Japan). Reverse transcription was performed at 37°C for 15 min, followed by 85°C for 5 s. PCR amplification was performed using cDNA templates and primers specific for 5'-untranslated region of HAV (sense primer, 5'-AGGCTACGGGTGAAACCTCTTAG-3', and antisense primer, 5'-GCCGCTGTTACCCTATCCAA-3'), for glucose-regulated protein 78 (GRP78) (sense primer, 5'-GCCTGTATTTCTAGACCTGCC-3', and antisense primer, 5'-TTCATCTTGCCAGCCAGTTG-3'), and for glyceraldehyde-3-phosphate dehydrogenase (GAPDH) (sense primer, 5'-ACCCACTCCTCCACCTTTG-3', and antisense primer, 5'-CTCTTGTGCTCTTGCTGGG-3') 9. Real-time PCR was performed with Power SyBr Green Master Mix (Applied Biosystems, Thermo Fisher Scientific, Inc., Waltham, MA, USA) and a StepOne Real-time PCR system (Applied Biosystems). The PCR reaction was performed as follows: 95 °C for 10 min, followed by 40 cycles of 95 °C for 15 s and 60 °C for 1 min. Data analysis was based on the ddCt method.

### Statistical analysis

Data are expressed as the mean ± standard deviation (SD). Statistical analysis was performed using Student's t-test. Results with p < 0.05 were considered statistically significant.

## Results

### HBV replication is inhibited in HepG2.2.15 cells superinfected with HAV compared to HepG2.2.15 cells not infected with HAV

**Figure 1:**
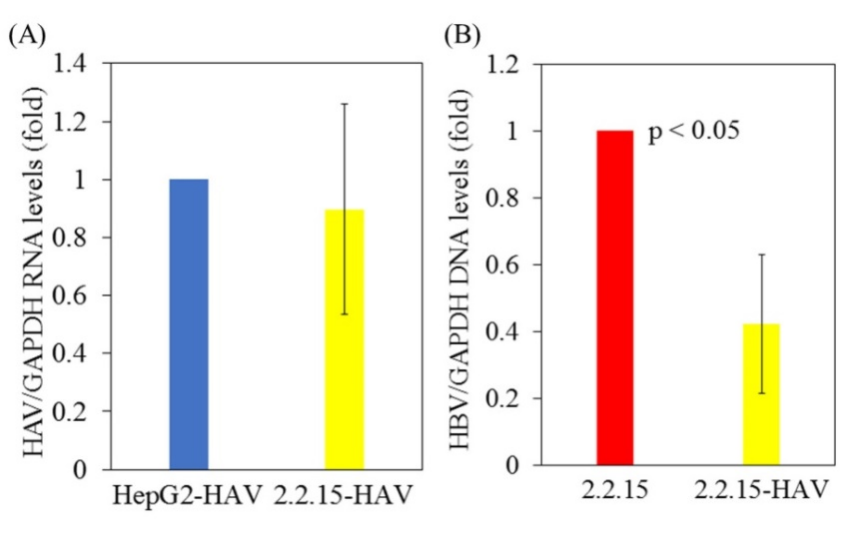
** HBV replication is inhibited in HAV-infected HepG2.2.15 cells compared to HepG2.2.15 cells. (A)** HAV RNA levels in HepG2 and HepG2.2.15 cells at 96 h post infection with the HAV HA11-1299 genotype IIIA strain. **(B)** HBV DNA levels in HepG2.2.15 cells infected with or without the HAV HA11-1299 genotype IIIA strain at 96 h post infection. HAV RNA and HBV DNA levels were measured by real-time RT-PCR. Data are presented as the mean ± SD of three independent experiments. *p < 0.05 compared to the untreated control.

First, HepG2 and HepG2.2.15 cells stably expressing HBV DNA 10, were infected with or without the HAV HA11-1299 strain. After 96 hours of infection, cellular RNA was extracted, and HAV RNA and HBV DNA levels were measured by real-time PCR. There was no difference in HAV RNA levels between HepG2 and HepG2.2.15 cells after HAV infection (Figure 1A). Interestingly, HBV DNA levels were reduced in HepG2.2.15 cells superinfected with HAV compared to HepG2.2.15 cells not infected with HAV (Figure 1B).

### Replication of both HAV and HBV is inhibited in PXB cells superinfected with HAV and HBV compared to cells monoinfected with HAV or HBV

**Figure 2:**
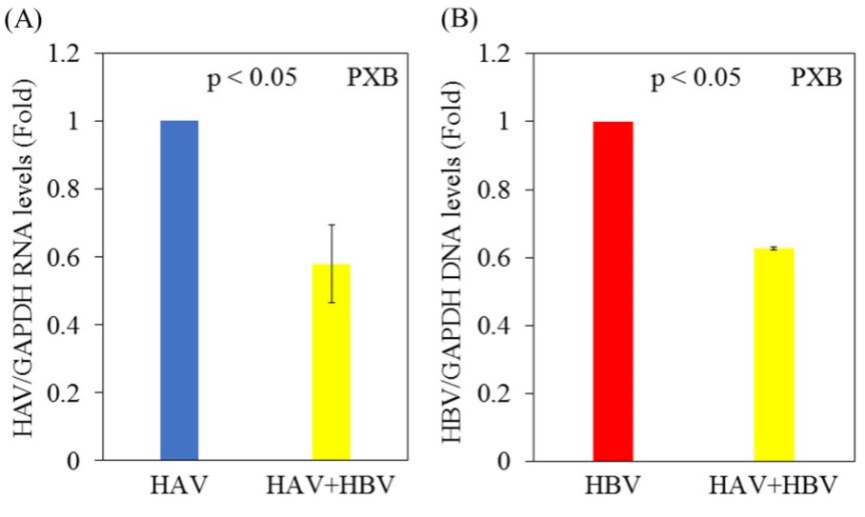
** Replication of both HAV and HBV is inhibited in PXB cells superinfected with HAV and HBV compared to those monoinfected with HAV or HBV. (A)** HAV RNA levels in PXB cells infected with HAV only and those superinfected with HAV and HBV genotype C. After 5 days of HBV infection, PXB cells were infected with the HAV HA11-1299 strain. Three days after infection with HAV, cellular RNA was extracted, and HAV RNA levels were measured using real-time PCR. **(B)** HBV DNA levels in PXB cells infected with HBV alone and those superinfected with HAV and HBV. After 5 days of HBV infection, PXB cells were infected with the HAV HA11-1299 strain. Three days after infection with HAV, cellular RNA was extracted, and HBV DNA levels were measured using real-time PCR. Data are presented as the mean ± SD of three independent experiments. *p < 0.05 compared to the untreated control.

Next, we analyzed the effects of HAV and/or HBV infection on HAV or HBV replication in PXB cells. PXB cells were initially infected with or without HBV genotype C and infected 5 days later with the HAV HA11-1299 strain. Three days after infection with HAV, cellular RNA was extracted, and HAV RNA and HBV DNA levels were measured by real-time PCR. Notably, HAV RNA levels were decreased in PXB cells superinfected with HAV and HBV compared with PXB cells infected with HAV alone (Figure 2A). HBV DNA levels were also lower in PXB cells superinfected with HAV and HBV compared with PXB cells infected only with HBV (Figure 2B).

Because our previous study demonstrated that GRP78 function as an antiviral agent against HAV 9, we also examined GRP78 expression in this superinfection model. Real-time RT-PCR showed that GRP78 expression in PXB cells superinfected with HAV and HBV was 1.19-fold higher than that in HAV-monoinfected PXB cells (n=3, P<0.05). However, there was no significant difference in GRP78 expression between PXB cells superinfected with HAV and HBV and PXB cells monoinfected with HBV.

### Inhibitory effects of Japanese rice-koji miso extracts on HAV replication in HepG2.2.15 cells infected with the HAV HA11-1299 strain

**Figure 3:**
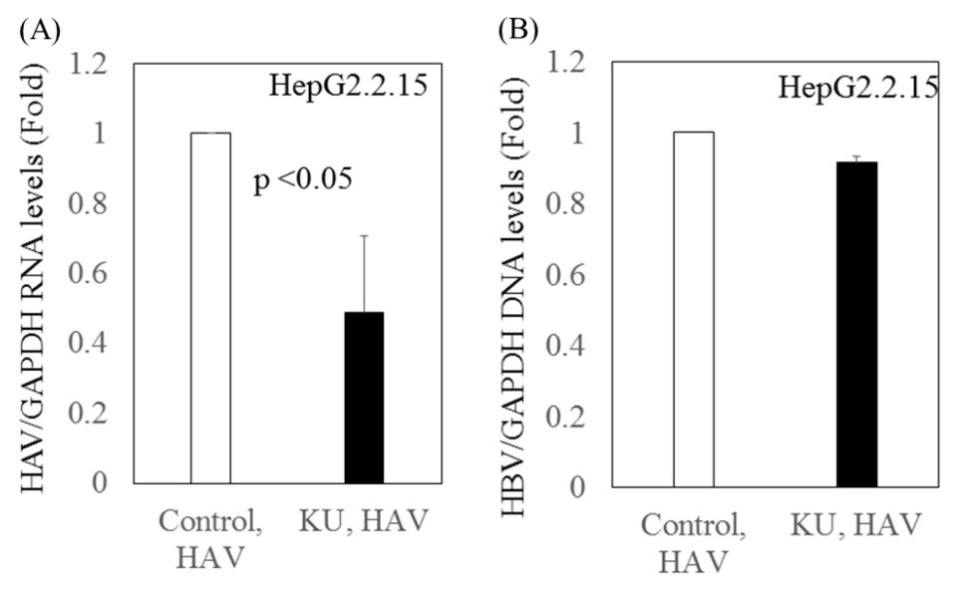
** Inhibitory effects of Japanese rice-koji miso extracts on HAV replication in HepG2.2.15 cells infected with HAV. (A)** HAV RNA levels **(B)** HBV DNA levels. After 72 hours of HAV HA11-1299 strain infection, cells were treated or not with Japanese rice-koji miso extracts [0.5% Kurasaigetsuusujiomiso (KU), Ando Brewery Kakunodate, Japan]. After 96 hours of infection, total cellular RNA and DNA were extracted for further analysis. HAV RNA and HBV DNA levels were measured using real-time PCR. Data are presented as the mean ± SD of three independent experiments. *p < 0.05 compared to the untreated control.

We previously reported that Japanese rice-koji miso extracts (0.5%Kurasaigetsuusujiomiso) enhance GRP78 expression and inhibit HAV replication in human hepatocytes 9. In the present study, we examined the effect of miso extracts on virus replication in HepG2.2.15 cells infected with the HAV HA11-1299 strain. Although the miso extracts had inhibitory effects on HAV replication, no impact on HBV replication was observed (Figure 3).

## Discussion

HepG2.2.15 cells are derived from HepG2 cells transfected with a plasmid carrying HBV DNA, producing HBV that caused hepatitis in chimpanzees 12, 13. In HAV-infected HepG2.2.15 cells, HBV replication was inhibited compared to in HepG2.2.15 cells not infected with HAV. Nonetheless, we observed no difference in HAV RNA levels between HepG2 and HepG2.2.15 cells after they were infected with HAV.

In search for a suitable cell-culture model for the analysis of superinfection of HAV with HBV, we identified PXB cells, which support both HAV and HBV replication 8, 9. In HBV-infected PXB cells superinfected with HAV, HBV replication was reduced compared to that in PXB cells infected with HBV alone. Thus, the present study demonstrated that to a certain extent, HAV infection inhibits HBV replication in two different cell culture models. Our results support previous reports 7, 14 that HAV infection also downregulates the expression of two HBV proteins in PLC/PRF/5 cells.

In HBV-infected PXB cells superinfected with HAV, HAV replication was inhibited compared to that in PXB cells infected only with HAV. PXB cells are primary hepatocytes, derived from a PXB mouse, a chimeric mouse with a humanized liver that is repopulated by human hepatocytes 8. As PXB mice were originally derived from albumin promoter/enhancer-driven urokinase-type plasminogen activator transgenic/severe combined immunodeficiency disease (uPA/SCID) mice, it is possible that PXB cells may be partly immunodeficient 15. Further studies will be needed to resolve this issue.

We also observed the inhibitory effects of Japanese rice-koji miso extracts on HAV replication in HepG2.2.15 infected with HAV. This finding supports our previous result that Japanese rice-koji miso extracts inhibit HAV replication. However, we did not observe inhibitory effects on HBV replication in the superinfection model of HepG2.2.15 cells infected with HAV.

HAV infections account for most of these cases, with 3% of jaundiced children shown to have acute HBV infection in Mongolia 16. It has been reported that HAV infection may have a severe clinical course in patients with underlying chronic liver disease, particularly among older individuals 17, and mortality from HAV infection increases in patients with chronic HBV infection 18. Despite effective vaccines for HAV and HBV infections, further studies are warranted to determine the mechanism by which Japanese rice-koji miso extracts inhibit HAV replication.

In conclusion, although there are several reports that HAV infection may have a severe clinical course in chronic HBV-infected patients superinfected with HAV, we found that HAV infection inhibited HBV replication. Japanese rice-koji miso extracts may have inhibitory effects on HAV replication in patients superinfected with HAV and HBV.

## References

[1] Vento S, Garofano T, Renzini C (1998). Fulminant hepatitis associated with hepatitis A virus superinfection in patients with chronic hepatitis C. N Engl J Med.

[2] Spada E, Genovese D, Tosti ME (2005). An outbreak of hepatitis A virus infection with a high case-fatality rate among injecting drug users. J Hepatol.

[3] Zhang X, Ke W, Xie J (2010). Comparison of effects of hepatitis E or A viral superinfection in patients with chronic hepatitis B. Hepatol Int.

[4] Sainokami S, Abe K, Ishikawa K (2005). Influence of load of hepatitis A virus on disease severity and its relationship with clinical manifestations in patients with hepatitis A. J Gastroenterol Hepatol.

[5] Sarin SK, Kumar M, Lau GK (2016). Asian-Pacific clinical practice guidelines on the management of hepatitis B: a 2015 update. Hepatol Int.

[6] Gauss-Müller V, Frösner GG, Deinhardt F (1981). Propagation of hepatitis A virus in human embryo fibroblasts. J Med Virol.

[7] Berthillon P, Crance JM, Leveque F (1996). Inhibition of the expression of hepatitis A and B viruses (HAV and HBV) proteins by interferon in a human hepatocarcinoma cell line (PLC/PRF/5). J Hepatol.

[8] Sasaki R, Kanda T, Wu S (2015). Association between hepatitis B virus and MHC class I polypeptide-related chain A in human hepatocytes derived from human-mouse chimeric mouse liver. Biochem Biophys Res Commun.

[9] Win NN, Kanda T, Nakamoto S (2018). Inhibitory effect of Japanese rice-koji miso extracts on hepatitis A virus replication in association with the elevation of glucose-regulated protein 78 expression. Int J Med Sci.

[10] Sasaki R, Kanda T, Nakamura M (2016). Possible Involvement of Hepatitis B Virus Infection of Hepatocytes in the Attenuation of Apoptosis in Hepatic Stellate Cells. PLoS One.

[11] Basu A, Kanda T, Beyene A (2007). Sulfated homologues of heparin inhibit hepatitis C virus entry into mammalian cells. J Virol.

[12] Sells MA, Chen ML, Acs G (1987). Production of hepatitis B virus particles in Hep G2 cells transfected with cloned hepatitis B virus DNA. Proc Natl Acad Sci U S A.

[13] Acs G, Sells MA, Purcell RH (1987). Hepatitis B virus produced by transfected Hep G2 cells causes hepatitis in chimpanzees. Proc Natl Acad Sci U S A.

[14] Gauss-Müller V, Deinhardt F (1984). Effect of hepatitis A virus infection on cell metabolism *in vitro*. Proc Soc Exp Biol Med.

[15] Ohshita H, Tateno C (2017). Propagation of Human Hepatocytes in uPA/SCID Mice: Producing Chimeric Mice with Humanized Liver. Methods Mol Biol.

[16] Ijaz S, Khulan J, Bissett SL (2006). A low rate of hepatitis B virus vaccine breakthrough infections in Mongolia. J Med Virol.

[17] Pramoolsinsap C (2000). Acute hepatitis A and acquired immunity to hepatitis A virus in hepatitis B virus (HBV) carriers and in HBV- or hepatitis C virus-related chronic liver diseases in Thailand. J Viral Hepat.

[18] Keeffe EB (1995). Is hepatitis A more severe in patients with chronic hepatitis B and other chronic liver diseases?. Am J Gastroenterol.

